# Exploring the role of migrasomes in myocardial injury and repair: A novel mechanism of mitochondrial quality control

**DOI:** 10.1016/j.gendis.2025.101639

**Published:** 2025-04-11

**Authors:** Chunnian Ren, Dawei He, Shulei Fan, Quan Wang

**Affiliations:** a Department of Cardiothoracic Surgery, Children’s Hospital of Chongqing Medical University, National Clinical Research Center for Child Health and Disorders, Ministry of Education Key Laboratory of Child Development and Disorders, Chongqing 400014, China; b Department of Respiratory Medicine, Second Affiliated Hospital of Chongqing Medical University, Chongqing 400010, China; c Chongqing Key Laboratory of Structural Birth Defect and Reconstruction, Chongqing 400014, China; d Department of Pediatric Surgery, Chengdu Women’s and Children’s Central Hospital, School of Medicine, University of Electronic Science and Technology of China, Chengdu 611731, China

A recent study suggests that low-intensity pulsed ultrasound selectively eliminates damaged mitochondria by promoting migrasome formation during myocardial ischemia-reperfusion injury, thereby enhancing mitochondrial quality control and reducing cardiomyocyte damage.^1^ This discovery first proposes the specific role and mechanism of migrasomes in the heart and provides preliminary evidence for their protective function against cardiomyocyte damage.

Migrasome, a newly identified organelle discovered in 2015, is implicated in migrasome-mediated mitocytosis, a novel mechanism of mitochondrial quality control.^2^ Its role in the heart, which is the most metabolically active human organ in terms of mitochondrial metabolism, has previously been elusive. Sun et al reported the first identification of a potential migrasome mechanism in myocardial ischemia-reperfusion injury within the cardiomyocyte cell line AC16.^1^ Our study, building on this, observed migrasome in chronic hypoxia conditions in the H9c2 cardiomyocyte cell line.^3^ These findings suggest that migrasome may play a broad role in myocardial injury (Fig. 1). Additionally, markers associated with migrasome have been correlated with the diagnosis of acute myocardial infarction,^4^ highlighting their clinical significance.

**Figure 1:**
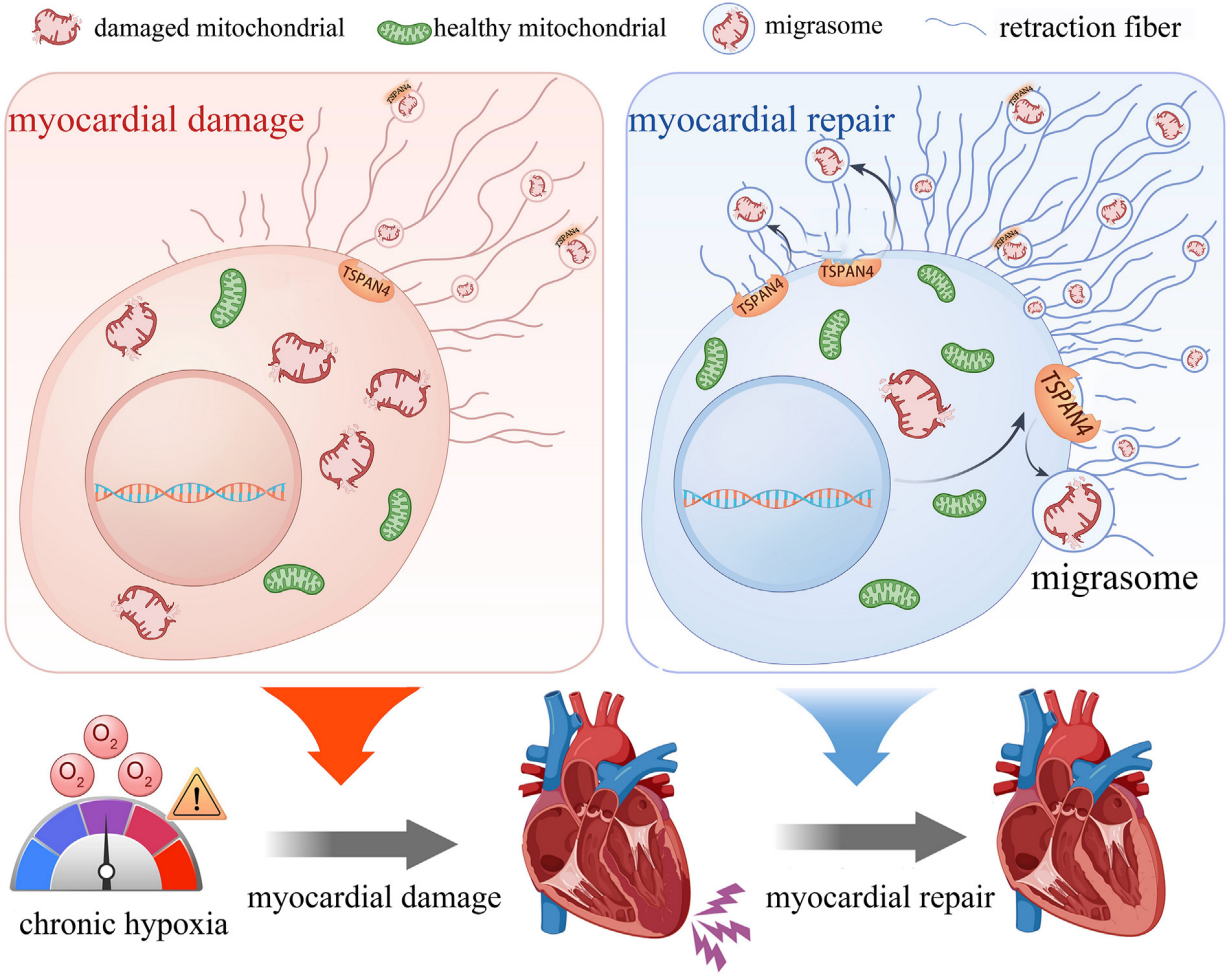
Under chronic hypoxia, migrasome-mediated mitocytosis is initiated in injured cardiomyocytes, regulating mitochondrial homeostasis by promptly removing damaged mitochondria and thereby facilitating the repair of cardiomyocyte injury.

However, we must emphasize that existing studies have only confirmed the presence of migrasomes in the AC16 *in vitro* cardiomyocyte cell line,^1^ and no *in vivo* studies have yet detected migrasomes in myocardial tissues. Moreover, no published studies have thus far provided direct evidence for the presence of migrasomes in primary cardiomyocytes. Given that AC16 cells are a rat-derived, immortalized cell line, they may not accurately represent *in vivo* cardiomyocytes in the context of cardiac diseases and pathological states. Consequently, the current research findings cannot be directly correlated with *in vivo* cardiomyocytes, highlighting a significant gap in the field. Furthermore, Sun et al employed a mouse model of cardiomyocyte-specific TSPAN4 (tetraspanin 4; a core functional protein of the migrasome^2^) knockout to investigate the role of the migrasome in myocardial ischemia-reperfusion injury.^1^ They did not conclusively demonstrate that the migrasome is a key mediator of low-intensity pulsed ultrasound-induced cardioprotection, and there is no direct evidence for the migrasome’s presence in cardiac tissues and primary cardiomyocytes. Thus, future studies are required to investigate the presence of migrasomes in cardiac tissue *in vivo*, including analyses of both cardiac tissue and primary cardiomyocytes.

Notably, the functionality of migrasome is contingent upon cell migration associated with the extracellular matrix. *In vivo*, the migratory capabilities and plasticity of cardiomyocytes may be significantly constrained due to the unique structure of mature cardiomyocytes and their interactions with one another. Concurrently, other cardiac cell types, such as fibroblasts and vascular endothelial cells, exhibit a higher degree of migratory capacity and dynamic cell membrane and cytoskeletal changes. Previous studies have identified the presence of migrasome in fibroblasts and vascular endothelial cells in various tissues.^2^ Therefore, even if migrasome is detected in cardiac tissue, its function may not be primarily derived from cardiomyocytes. Future studies employing single-cell RNA sequencing of cardiac tissues will be essential to pinpoint the specific cell types and molecular mechanisms underlying the function of migrasome in cardiac tissues.

The mitochondrial quality control mechanisms within cardiomyocytes are intricate, with autophagy traditionally recognized as a significant contributor.^5^ However, recent research has revealed that autophagy is primarily among the mechanisms cardiomyocytes employ in response to severe stimuli, such as myocardial ischemia-reperfusion injury.^5^ Migrasome-mediated mitocytosis, initiated under mild stress, efficiently removes damaged mitochondria, avoids the accumulation of intracellular degradation byproducts, and maintains mitochondrial homeostasis at the organismal level. Therefore, we hypothesize that autophagy and migrasome-mediated mitocytosis cooperatively modulate chronic hypoxic injury in cardiomyocytes and maintain homeostasis under varying stimulus intensities. Rho-associated coiled-coil containing protein kinase 1 (ROCK1) is known to regulate migrasome production through its effects on cytoskeletal dynamics and cell migration. This role is particularly relevant in the context of myocardial injury, as migrasomes may contribute to the removal of damaged mitochondria and the maintenance of mitochondrial quality control. However, in our previous studies, we found that ROCK1 could act as a direct binding site downstream of the transcription factor forkhead box O1 (FOXO1) in cardiomyocytes, directly affecting mitochondrial quality control and exacerbating cardiomyocyte damage.^3^ These studies highlight the complex regulation of ROCK1 in cardiomyocyte injury. Nevertheless, as we previously noted, given that the migratory capacity and plasticity of mature cardiomyocytes are significantly constrained, it remains unclear whether the presence of migrasomes in cardiomyocytes would indicate that their function in cardiac tissue is primarily derived from cardiomyocytes themselves. Therefore, future studies are warranted to further explore the relative significance of migrasome-mediated mitocytosis in cardiomyocyte injury compared with other mitochondrial quality control pathways.

In conclusion, the study by Sun et al has laid the foundation for novel investigations into the role of migrasomes within cardiac tissue. However, the current data are insufficient to definitively determine the true source of migrasome formation in cardiac tissue *in vivo* and the specific mechanisms by which migrasomes exert their functions. These gaps highlight the necessity for further research to explore the role of migrasomes in cardiac tissue. To this end, future studies may require researchers to conduct more comprehensive studies, including single-cell RNA sequencing of cardiac tissue and the detection of migrasomes in primary cardiomyocytes and cardiac tissue. Additionally, attention should be paid to the differences and underlying reasons for the presence of migrasomes in cardiomyocyte-related cell lines such as H9C2, AC16, and primary cardiomyocytes. These efforts aim to further elucidate the sources of migrasome formation in cardiac tissue *in vivo* and the functional mechanisms of migrasomes in cardiomyocyte injury and repair. The objective of these studies is to gain a deeper understanding of the functions and mechanisms of migrasomes in the context of cardiomyocyte injury and repair, thereby potentially revealing novel therapeutic perspectives for the treatment of cardiomyocyte damage.

## CRediT authorship contribution statement

**Chunnian Ren:** Writing – original draft. **Dawei He:** Writing – original draft. **Shulei Fan:** Writing – review & editing. **Quan Wang:** Writing – review & editing.

## Funding

This work was supported by the 10.13039/501100001809National Natural Science Foundation of China (No. 82400362) and the 10.13039/501100001809National Natural Science Foundation of China (No. 82200095).

## Conflict of interests

The authors declared no competing interests.
